# Blister-inducing antibodies target multiple epitopes on collagen VII in mice

**DOI:** 10.1111/jcmm.12338

**Published:** 2014-08-05

**Authors:** Kinga Csorba, Mircea Teodor Chiriac, Florina Florea, Miruna Georgiana Ghinia, Emilia Licarete, Andreea Rados, Alexandra Sas, Vlad Vuta, Cassian Sitaru

**Affiliations:** a Department of Dermatology, University of FreiburgFreiburg, Germany; b Faculty of Biology, University of FreiburgFreiburg, Germany; c Molecular Biology Center, Interdisciplinary Research Institute on Bio-Nano-Sciences and the Department of Biology, Babes-Bolyai UniversityCluj-Napoca, Romania; d Center for Biological Signalling Studies (BIOSS), University of FreiburgFreiburg, Germany

**Keywords:** autoimmunity, collagen VII, epidermal basement membrane, functional epitope mapping

## Abstract

Epidermolysis bullosa acquisita (EBA) is an autoimmune subepidermal blistering disease of mucous membranes and the skin caused by autoantibodies against collagen VII. *In silico* and wet laboratory epitope mapping studies revealed numerous distinct epitopes recognized by EBA patients' autoantibodies within the non-collagenous (NC)1 and NC2 domains of collagen VII. However, the distribution of pathogenic epitopes on collagen VII has not yet been described. In this study, we therefore performed an *in vivo* functional epitope mapping of pathogenic autoantibodies in experimental EBA. Animals (*n* = 10/group) immunized against fragments of the NC1 and NC2 domains of collagen VII or injected with antibodies generated against the same fragments developed to different extent experimental EBA. Our results demonstrate that antibodies targeting multiple, distinct epitopes distributed over the entire NC1, but not NC2 domain of collagen VII induce blistering skin disease *in vivo*. Our present findings have crucial implications for the development of antigen-specific B- and T cell-targeted therapies in EBA.

## Introduction

Collagen VII is the main constituent of the anchoring fibrils, structures that mediate the adhesion of the epidermis onto the dermis, and thus has a critical role in providing skin stability and integrity [1]. The essential role that collagen VII plays in the biology of the dermal–epidermal junction (DEJ) is exemplified by inherited or targeted disruptions in the gene that encodes it, which yield a phenotype characterized by subepidermal blisters [2–6]. Autoantibodies against collagen VII induce tissue damage in epidermolysis bullosa acquisita (EBA), an autoimmune chronic blistering disease of the skin and mucous membranes [7,8]. Interestingly, autoantibodies against collagen VII have been documented in patients with several autoimmune and inflammatory diseases, including pemphigus, bullous pemphigoid, inflammatory bowel disease and systemic lupus erythematosus [9–12]. However, the pathogenic relevance of these autoantibodies, which occur generally at lower reactivity when compared with EBA, has not yet been clearly demonstrated.

Collagen VII is a homotrimer composed of α chains consisting of a 145-kD central collagenous triple-helical fragment flanked by a large 145-kD amino-terminal, non-collagenous domain (NC1), and a small 34-kD carboxyl-terminal non-collagenous domain (NC2; Fig. S1A of the Supporting Information) [12,13]. After secretion into the extracellular space, collagen VII molecules form antiparallel, tail-to-tail dimers stabilized by disulphide bonding through a small carboxyl-terminal NC2 overlap, while a portion of the NC2 domain is proteolitically removed [13–15]. The NC1 domain consists of multiple subdomains including segments with homology to adhesion molecules, including the cartilage matrix protein (CMP), nine consecutive fibronectin type III-like repeats, and a segment with homology to the A domain of von Willebrand factor [16].

The pathogenic relevance of antibodies against collagen VII is supported by compelling evidence: (*i*) EBA autoantibodies were shown to recruit and activate leucocytes *ex vivo*, resulting in dermal–epidermal separation in cryosections of human skin [17]; (*ii*) antibodies against collagen VII induce subepidermal blisters when passively transferred into mice [8,18]; (*iii*) immunization with recombinant autologous collagen VII induces an autoimmune response to this protein, resulting in a blistering phenotype closely resembling human EBA [19,20] and (*iv*) the transfer of maternal antibodies against collagen VII induces transient blistering in the newborn [21]. Further studies using these *ex vivo* and animal models of EBA unravelled the mechanisms of tissue damage in experimental EBA, which require the activation of downstream inflammatory pathways [17,22–26].

Characterization of the epitopes targeted by pathogenic autoantibodies is crucial for the development of new antigen-specific therapies, including T- and B cell-directed immunomodulatory/depletive approaches or epitope-specific immunoadsorption of antibodies [27]. Epitope-distribution mapping studies with EBA patients' sera revealed that the major epitopes recognized by autoantibodies reside within the NC1 domain of native collagen VII [28,29]. In addition to very few cases showing reactivity to the triple-helical domain of collagen VII, further epitopes of EBA autoantibodies have been more recently mapped to the NC2 domain [12,30–32]. Immunization and antibody passive transfer studies have clearly demonstrated that blister-inducing autoantibodies target epitopes within the CMP domain (aa 1-227) and a stretch of 200 amino acids (aa 757-967) within the NC1 domain [8,19,33]. However, the pathogenic relevance of further epitopes recognized by patients' autoantibodies has not yet been addressed experimentally.

In this study, we therefore performed an *in vivo* functional epitope-distribution mapping, using previously established experimental models of EBA and five overlapping fragments (mCVII-1, aa 26-300; mCVII-2, aa 281-594; mCVII-3, aa 561-879; mCVII-4, aa 871-1125; and mCVII-5, aa 1108-1323) spanning the entire NC1 domain as well as a fragment (mCVII-z, aa 2795-2944) corresponding to the NC2 domain of murine collagen VII. These proteins were used to produce specific polyclonal antibodies in rabbits, and the purified IgG fractions specific to different regions of collagen VII were injected into wild-type BALB/c mice. The animals injected with antibodies against fragments of the NC1 domain developed to different extent experimental EBA. Antibodies against the NC2 domain similarly to the normal rabbit IgG used as control did not induce skin disease. In a further set of experiments, we immunized SJL mice with the different collagen VII fragments and observed them for several months. While collagen VII-specific autoantibodies were produced in all groups, clinical and histopathological disease mainly developed in mice immunized with fragments of the NC1 domain. Our results clearly demonstrate that antibodies targeting multiple, distinct epitopes distributed over the entire NC1 domain of collagen VII induce blistering skin disease *in vivo*. The present findings have crucial implications for the development of antigen-specific B- and T cell-targeted therapies in EBA.

## Materials and methods

### Mice

Six to 8-week-old BALB/c female mice with a bodyweight of ∼17 g, and 6–8 week-old SJL-1 female and male mice with a bodyweight of ∼20 g were obtained from Charles River Laboratories (Wilmington, MA, USA). All injections and bleedings were performed on mice narcotized by inhalation of isoflurane or s.c. administration of a mixture of ketamine (100 μg/g) and xylazine (15 μg/g). The experiments were approved by local authorities of the Animal Care and Use Committee (G-11/43; 31458/2010) and performed by certified personnel. While different wild-type mouse strains are susceptible for the induction of skin blistering by the passive transfer of collagen VII-specific autoantibodies [8], SJL mice are readily developing the autoimmune skin blistering by immunization with the autoantigen [19].

### Recombinant forms of murine collagen VII

The following overlapping fragments of the NC1 domain: mCVII-1 (aa 26-300), mCVII-2 (aa 281-594), mCVII-3 (aa 561-879), mCVII-4 (aa 871-1125), mCVII-5 (aa 1108-1323), mCVII-Cr (aa 759-980) and the mCVII-z (aa 2795-2944) fragment corresponding to the NC2 domain of murine collagen VII were expressed as glutathione-S-transferase (GST) fusion and His-tagged proteins in *Escherichia coli* as previously described (Fig. S1A of Supporting information) [34]. The full-length form of murine collagen VII was obtained following published protocols [34]. Briefly, the cDNA sequences coding for three fragments of the NC1 and two fragments corresponding to the collagenous and NC2 domains of murine collagen VII were cloned into pcDNA5/FRT vector and expressed in Flp-In HEK 293T human embryonic kidney cells (Flp-In™-293; Invitrogen, Carlsbad, CA, USA). The construction of entire cDNA sequence utilized the overlapping internal restriction sites including NheI, Asp, AgeI, AvrII and AarI from each cDNA fragment. DNA sequence data for murine collagen VII were retrieved from GenBank using the accession number NM_007738 http://www.ncbi.nlm.nih.gov/gene/?term=NM_007738. Seventy per cent confluent Flp-In HEK 293T human embryonic kidney cells were transfected with 3 μg of recombinant vector. When they reached 90% confluency, they were grown in serum-free DMEM medium, without phenol red (Gibco, Darmstadt, Germany) supplemented with L-glutamine, penicillin, streptomycin, hygromycin (all from Biochrome, Berlin, Germany) and 100 μg/ml vitamin C. Two days after medium change and vitamin C addition, the medium was collected, centrifuged, PMSF and EDTA were added to a final concentration of 0.1 and 0.5 M respectively, and stored at −20°C. The recombinant murine collagen VII full length was concentrated from the harvested culture media by 30% ammonium sulphate precipitation, as described [12].

### Immune rabbit sera

Two to four New Zealand White rabbits/mCVII fragment and GST (mCVII-1, mCVII-2, mCVII-3, mCVII-4, mCVII-5, mCVII-Cr, mCVII-z) were immunized s.c. with 200 μg of purified, recombinant protein in Freund's complete adjuvant. The animals received two boosting injections with the same protein amount suspended in incomplete Freund's adjuvant at 2 weeks intervals, except for the rabbits of the speedy immunization programme who received boosts 7, 10 and 18 days after the first injection. Immune sera were obtained at regular intervals and characterized by immunofluorescence (IF) microscopy on cryosections of murine skin. Normal rabbit IgG (NRIgG) was obtained from pre-immune rabbit serum.

### Affinity purification of IgG

Total IgG from immune and normal rabbit serum was isolated using Protein G Sepharose 4 Fast Flow affinity column chromatography (GE Healthcare, Freiburg, Germany). Specific antibodies to mCVII-5 and mCVII-z were additionally affinity purified on AminoLink immobilization kit following manufacturer's instructions (Thermo Scientific Pierce, Rockford, IL, USA). Briefly, GST-mCVII-5 and GST-mCVII-z recombinant proteins were covalently coupled to 4% beaded agarose at pH 10 and the sera were incubated with protein-coupled matrix. Antibodies were eluted with 0.1 M glycine buffer (pH 2.5), neutralized with 1.5 M Tris-HCl (pH 10), and concentrated under extensive washing with PBS (pH 7.2) using Amicon Ultra-15 filters (Millipore, Darmstadt, Germany). The concentration of the purified IgG was measured spectrophotometrically at 280 nm. Reactivity and end-point titres of the purified IgG solutions were analysed by indirect IF microscopy on salt-split murine skin [8]. For the passive transfer into mice, the IgG preparations were adjusted to similar reactivity, *i.e*. end-titres as measured by indirect IF microscopy on skin sections.

### Induction of experimental EBA

Experimental EBA was induced by active immunization using collagen VII fragments or by passive transfer of collagen VII-specific antibodies, as described [8,19], with minor modifications. Briefly, for inducing a collagen VII-specific autoimmune response and skin blistering, SJL-1 mice (*n* = 10/group) were immunized s.c. at the tail's base and hind foot pads and boosted after 3, 6 and 9 weeks with 50 μg of either individual fragments of recombinant murine collagen VII (GST-mCVII-1, GST-mCVII-2, GST-mCVII-3, GST-mCVII-4, GST-mCVII-5, GST-mCVII-z) or an equimolar mixture of these (test groups 1–7), or our control protein GST (control group 8, respectively) emulsified in the non-ionic block copolymer adjuvant TiterMax (Sigma-Aldrich, Seelze, Germany). Mice were weighed and evaluated every second day for their general condition and every week for clinical signs of EBA, such as erythema, blisters, thickening of the ears, scarring, erosions and alopecia. The extent of the disease activity was determined by quantifying the affected skin surface (see scoring system below). Mice were bled before the first immunization and every second week thereafter for a period of 12 weeks. Serum was tested for autoantibody titres and isotype distribution. Biopsies of lesional and perilesional skin were collected after the euthanasia of mice, and subsequently prepared for IF microscopy and histopathology [35].

For the passive transfer of IgG specific to fragments of murine collagen VII, we injected mice with preparations having the same reactivity against the DEJ in the adequate volumes. Repeated injections with decreasing volumes were administered every second day, four times (see Table 1). Exception to this injection schedule were the mice receiving antibody preparations against mCVII-5 and mCVII-z, as these preparations had a higher IgG concentration, we had to limit the number of injections to 3. The injected amounts (mg) of purified IgG against GST and normal rabbit IgG (NRIgG) were equivalent to the highest injected amount of collagen VII-specific IgG. Daily, mice were weighed, their ear thickness was measured using a calliper and examined for their general condition and for evidence of cutaneous lesions. Intact blisters, erosions or crusts and alopecia were quantified and the extent of skin disease was scored as follows: 0, no lesions; 1, less than 1% of the skin surface; 2, 1–5% of the skin surface; 3, 5–10% of the skin surface; 4, 10–20% of the skin surface affected. Weight loss of 5–10% of the total bodyweight during three consecutive days counts as an extra point in the final score. Every second day, blood was drawn and sera were obtained to assay the antibody titres by IF microscopy. Biopsies of lesional and perilesional skin, oesophagus, snout and lip were obtained 6 days after the last injection and were set up for histopathology and IF microscopy.

**Table 1:** Passive transfer of purified rabbit IgG

	Mice/group	Ab titre	Volume of injected IgG (μl)			
	
			1st injection	2nd injection	3rd injection	4th injection
IgG α-mCVII-1	*n* = 10	1:100000	375	250	250	125
IgG α-mCVII-2	*n* = 10	1:128000	300	200	200	100
IgG α-mCVII-3	*n* = 10	1:128000	300	200	200	100
IgG α-mCVII-4	*n* = 10	1:128000	300	200	200	100
IgG α-mCVII-5	*n* = 6	1:3200	900	600	600	-
IgG α-mCVII-z	*n* = 5	1:3200	900	600	600	-
IgG α-GST	*n* = 8	0	200	130	130	100*
NR IgG	*n* = 8	0	270	170	170	100*

* Stock IgG solution was diluted to inject the volume in 100 μl (less would have been impractical).

### Immunofluorescence microscopy and immunoblot analysis

Serum and tissue bound rabbit or mouse autoantibodies were detected following published protocols with minor modifications [8]. Briefly, after incubating with serial dilutions of rabbit immune sera or fragment-specific affinity-purified total rabbit IgG, the frozen sections of normal and/or salt-split murine skin were treated with 100-fold diluted, AlexaFluor 488-conjugated Abs to rabbit IgG (Invitrogen, Molecular Probes®, Darmstadt, Germany). Linear deposition on the dermal side of the artificial split was seen for the antibodies against the collagen VII fragments but not for GST or NRIgG (Fig. S2A of Supporting Information). Direct IF microscopy was performed on frozen sections, obtained from murine tissue biopsies, using 100-fold and 40-fold diluted AlexaFluor 488-labelled Abs specific to rabbit or mouse IgG and murine C3 respectively (all from Invitrogen, Molecular Probes®). The staining intensity of immunoreactants in the skin of immunized mice was assessed by measuring the total fluorescence of the selected DEJ area and expressed as mean fluorescence density arbitrary units on a linear scale of 0–256 and in non-saturating condition of the Nikon ECLIPSE 80i (DS-Fi1) (Duesseldorf, Germany) camera (filter: UV-1 EX365/10 DM400 AM400, software: NIS Elements F4.00.06), using the ImageJ software (http://imagej.nih.gov/ij/) or/and semi-quantitatively using a score comprising of 0, for no staining; 1, faint staining; 2, medium; and 3, intense staining (19). As previously described [34], the complement-fixing activity of antibodies to the DEJ was determined by incubating sections of normal mouse skin with diluted immune rabbit sera against fragments of collagen VII or NRS at RT. In a second step, fresh human serum as a source of complement, diluted 1:5 in Gelatin Veronal Buffer (Sigma-Aldrich) was added to the sections. *In situ* complement deposition was visualized using a 100-fold diluted FITC-conjugated antibody specific for human C3 (MPBiologicals, Santa Ana, CA, USA). For immunoblotting, recombinant proteins were fractionated by 12% SDS-PAGE and transferred to nitrocellulose. The specific binding of the 200-fold diluted immune rabbit sera was detected with an HRP-conjugated rabbit IgG-specific Ab (NOVUS Biologicals; Fig. S2B of Supporting Information).

### ELISA

To measure the serum levels of rabbit and mouse collagen VII-specific antibodies, ELISA was performed as described, with minor modifications [20,23,36]. Briefly, microtitre plates (Greiner Bio-One, Frickenhausen, Germany) were coated, overnight with 200 or 500 ng of recombinant full-length collagen VII protein in 0.1 M bicarbonate buffer (pH 9.6). After blocking, wells were incubated with a 250-fold or a 50-fold dilution of mouse sera. Bound Abs were detected using horseradish peroxidase (HRP)-conjugated 3000 times or 2000 times diluted secondary Abs specific for rabbit (Novus Biologicals, Littleton, CO, USA) and mouse IgG (Invitrogen, Molecular Probes®) respectively, and orthophenylene diamine (Dako, Hamburg, Germany) as a chromophore (492 nm). For IgG subclass identification, the incubation with the 100 times diluted mouse sera was followed by incubation with 250 times diluted biotinylated monoclonal rat antimouse IgG1, IgG2a, IgG2b and IgG3 Abs (all from BD Pharmingen, Heidelberg, Germany), and HRP-conjugated streptavidine (Dianova, Hamburg, Germany). The immunoreactions were visualized as described above.

### *Ex vivo* antibody-induced dermal–epidermal separation

The ability of immune rabbit sera to activate human leucocytes was assessed using an *ex vivo* assay of antibody-induced granulocyte-dependent dermal–epidermal separation in cryosections of normal skin [37]. IgG specific for collagen VII fragments was incubated with cryosections of mouse skin. The extent of dermal–epidermal separation was scored as follows: 1, if 1–33%; 2, if 33–66%; 3, if 66–100% of the length of the DEJ is separated. For the experiments conducted with human leucocytes, we obtained approval from the Ethics Committee of the Medical Faculty of the University of Freiburg, Germany (Institutional Board Project no. 407/08). Written informed consent was obtained from donors whose material was used in the study, in adherence to the Helsinki Principles.

### Histology

Biopsies of lesional skin and oesophagus as well as sections from the *ex vivo* cryosection assay were fixed in 3.7% buffered formalin. Sections from paraffin-embedded tissues were stained with haematoxylin and eosin.

### MPO assay

Neutrophil infiltration of murine skin was assessed as described [25] with minor modifications. Briefly, the left ear of clinically diseased and not diseased mice was removed after euthanasia and extracts were prepared by homogenizing with the Ultra turrax dispenser (Ika, Sigma-Aldrich) in a 0.1 M Tris-Cl, pH 7.6, 0.15 M NaCl, 0.5% hexadecyl trimethylammoniumbromide (Sigma-Aldrich) containing buffer. Myeloperoxidase (MPO) activity in the supernatant fraction was measured by the change in optical density at 460 nm resulting from decomposition of H_2_O_2_ in the presence of o-dianisidine (Sigma-Aldrich). A standard reference curve was established using known concentrations of purified MPO (Sigma-Aldrich). MPO content was expressed as units of MPO activity per mg of protein. Protein concentrations were determined by the BCA assay (Thermo Scientific).

### Statistical analysis

Analyses were performed with the GraphPad Prism statistical package (version 5.03). Continuous variables were compared using the Man–Whitney U or Student's *t*-test. The Kruskal–Wallis test was usually followed by Dunn's multiple comparison test, when more than three groups were analysed. anova and Dunnett's test were used to compare the data having normal distribution. Correlation analysis was done using the Spearman's rank correlation test. *P* < 0.05 was considered statistically significant.

## Results

### Passively transferred pathogenic antibodies target multiple epitopes on the NC1 domain of collagen VII

Balb/c mice were injected with IgG purified from immune rabbit sera raised against collagen VII fragments (Table 1). The injected collagen VII-specific IgG preparations showed similar reactivity against the DEJ as measured by the end-point titre by indirect IF microscopy (Fig. S2A of Supporting Information). Control mice received injections of IgG purified from pre-immune sera or from rabbits immunized with GST.

**Fig. 1:**
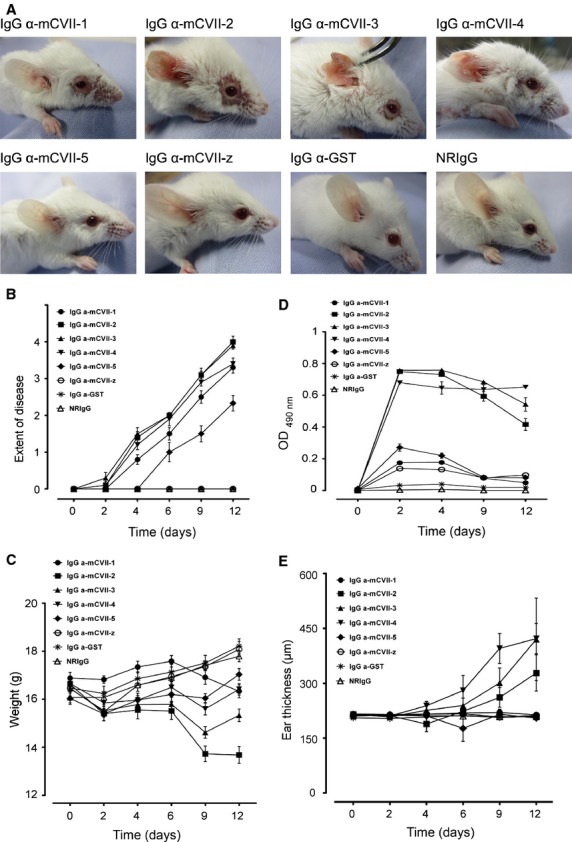
Disease phenotype and severity of experimental epidermolysis bullosa acquisita induced by the passive transfer of collagen VII-specific IgG. (**A**) BALB/c mice received injections of IgG purified from rabbits immunized with different glutathione-S-transferase (GST)-fusion proteins containing fragments of murine collagen VII (mCVII-1, *n* = 10; mCVII-2, *n* = 10; mCVII-3, *n* = 10; mCVII-4, *n* = 10; mCVII-5, *n* = 6; mCVII-z, *n* = 5) or GST alone (*n* = 8), or from pre-immune animals (*n* = 8). Mice injected with rabbit IgG against mCVII-1, mCVII-2, mCVII-3, mCVII-4 and mCVII-5, but not against mCVII-z and GST or injected with pre-immune IgG, developed skin blisters and erosions covered by crusts. (**B**) The disease severity, based on a scoring system (4 being the highest score) and depicted as means of individual clinical scores ± SEM is shown before the first injection and every other following day for 12 days. Significantly more extensive disease was induced by injecting IgG against fragments mCVII-2, mCVII-3, mCVII-4 and mCVII-1, *P* < 0.05 *versus* GST and NRIgG (Kruskal–Wallis test followed by Dunn's test). (**C**) Time course for mean weight ± SEM shows an increasing tendency in case of mice injected with IgG against mCVII-z, GST and NRIgG, whereas the most significant weight loss is seen in mice receiving IgG against mCVII-3 and mCVII-2, *P* < 0.05 *versus* mCVII-z, GST and NRIgG, *n* = 8 (Kruskal–Wallis test followed by Dunn's test). (**D**) Serum levels of collagen VII fragment-specific or control rabbit IgG were measured by ELISA. Mean OD readings ± SEM for the mice groups injected with mCVII-2, mCVII-3 and mCVII-4 showed higher levels of Abs in comparison to the rest of the groups. (**E**) Thickness of the same ear was measured before the first injection as well as every second day, for 12 days. Results are shown as means of individual measurements ± SEM.

Three or 4 days after the first injection, mice that received IgG against collagen VII fragments mCVII-2, mCVII-3 and mCVII-4 started to show alopecia around the swollen eyes and in the mCVII-4-specific IgG-injected group on the trunk. Single blisters on their ears were preceded and accompanied by mild erythema. Extensive widespread lesions such as erosions and crusts with or without alopecia developed 5–10 days after the first antibody administration (Fig. 1A). Mice from the groups receiving antibodies against fragments mCVII-2 and mCVII-3 showed extensive involvement of the head and neck area, with haemorrhagic blisters and erosions, crusts and alopecia around their eyes, on their snout, lips and ears (see Fig. S1B of Supporting Information). Lesions on the limbs and on the back were also present, to a lesser extent. In contrast to the previously described groups, the animals injected with mCVII-1-specific IgG had less extensive disease on the head and neck, but had widespread alopecia on their dorsal and dorso-lateral body areas. The clinical scores showed no significant difference between these four groups (Fig. 1B). Mice receiving antibodies against mCVII-5 initially developed alopecia on their trunk sometimes with erosions or crust, but also had mild crusts and erosions on their snout, limbs and the base of the ears (Fig. 1A, Fig. S1B of Supporting Information). No skin lesions were seen in mice injected with antibodies against mCVII-z, GST or control IgG at any time during the observation period.

Mice receiving injections of mCVII-2- and mCVII-3-specific IgG showed significant weight loss (*P* < 0.001 *versus* control, *n* = 8, and *P* < 0.05 *versus* control, *n* = 10; Fig. 1C). Mice in the rest of the groups showed either a normal weight gain curve (mCVII-z, GST) like mice in the control group or maintained their initial body mass (mCVII-1, mCVII-4, mCVII-5) with slight fluctuations. By measuring the ear thickness, progressive ear swelling was observed especially in mice injected with mCVII-4- and mCVII-3-specific IgG (*P* < 0.05 *versus* control, *n* = 10 and *n* = 8; Fig. 1E).

Direct IF microscopy analysis performed on skin biopsies revealed linear deposition of rabbit IgG (Fig. S3A of Supporting Information) at the murine skin basement membrane in all experimental groups receiving antibodies against collagen VII fragments, and showed similar fluorescence intensity when this was quantified (Fig. 3A, Table S1 of Supporting Information). No deposits of rabbit IgG at the DEJ were seen in mice injected with GST-specific or pre-immune IgG.

### Immunization of mice with several fragments of the NC1 domain of collagen VII induces skin blistering disease

**Fig. 2:**
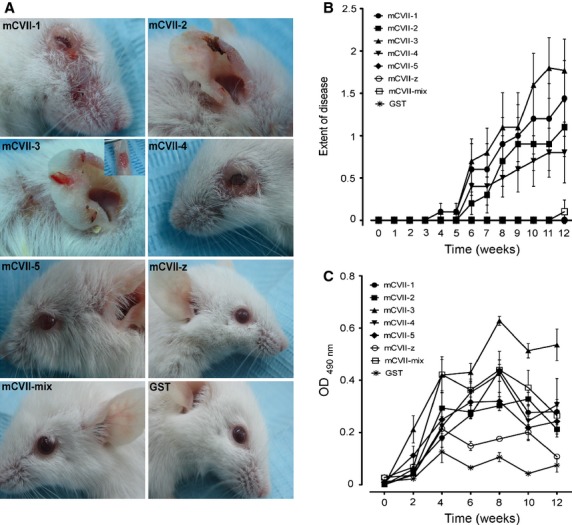
Disease phenotype and severity of experimental EBA induced by immunization with recombinant protein corresponding to different fragments of murine collagen VII. (**A**) SJL-1 mice (*n* = 10/fragment) immunized with an equimolar mix or individual proteins of recombinant collagen VII developed at different extent lesions on the skin including alopecia, blisters, erosions and crusts. Control mice immunized with GST (*n* = 10) had a normal appearance. (**B**) The disease severity, based on a scoring system (4 being the highest score) and depicted as means of individual clinical scores ± SEM is shown before the first injection and every following week for 12 weeks. Mice immunized with GST-mCVII-3 and GST-mCVII-1 showed the highest clinical scores, whereas less or no disease activity is seen in the other experimental groups. (**C**) Levels of collagen VII-specific mouse IgG in serum samples of mice immunized with recombinant forms of collagen VII (*n* = 10/fragment) or GST (*n* = 10) were measured by ELISA. Data are shown as mean OD reading ± SEM.

SJL-1 mice were randomly assigned to different groups (*n* = 10/group) and injected with either individual GST-fusion protein containing fragments of murine collagen VII or with an equimolar mixture of these proteins. Mice in the control group (*n* = 10) received GST as an antigen. Initial lesions appeared 4 weeks after the first immunization, around the eyes of mice immunized with GST-mCVII-1 and GST-mCVII-3. Mice challenged with GST-mCVII-1, -2, -3 and -4 developed lesions including blisters, erosions partly covered by crusts and alopecia 6 weeks after the first immunization. Over time, the extent of disease increased progressively in mice of these groups (Fig. 2A and B), whereas mice in the remainder groups, including those immunized with the mixture as well as mice in the GST control group did not show skin changes (Fig. 2A and B).

Direct IF microscopy analysis performed on skin biopsies revealed linear deposition of mouse IgG at the epidermal basement membrane in mice immunized with collagen VII fragments 1, 2, 3, 4 and the mix of peptides, and showed similar fluorescence intensity when this was quantified. No deposits of mouse IgG at the DEJ were seen in mice injected with GST-specific antibodies, and in the groups immunized with mCVII-5 and mCVII-z (Table S2 of Supporting Information).

### Serum levels of mCVII fragment-specific IgG antibodies correlate with disease activity

The levels of collagen VII-specific rabbit antibodies in the sera of BALB/c mice were measured by ELISA using recombinant full-length murine collagen VII and are depicted in Figure 1D. The serum levels of collagen VII fragment-specific antibodies correlated well with the extent of skin disease (*r*_s_ = 0.7, *P* < 0.0001, *n* = 67). The co-variation was more obvious when the circulating specific autoantibody level and the disease activity of individual groups were considered (Table 2).

**Table 2:** Correlation of serum levels of mCVII fragment-specific antibodies with disease activity in the passive transfer model of experimental EBA

	Spearman *r*	*P*-value	95% CI	XY pairs
IgG α-mCVII-1	*r* = 0.74	0.0004	0.4187–0.9030	*n* = 18
IgG α-mCVII-2	*r* = 0.7	0.001	0.3413–0.8849	*n* = 18
IgG α-mCVII-3	*r* = 0.83	<0.0001	0.6010–0.9399	*n* = 18
IgG α-mCVII-4	*r* = 0.82	<0.0001	0.5631–0.9328	*n* = 18
IgG α-mCVII-5	*r* = 0.84	<0.0001	0.5799–0.9496	*n* = 15

In SJL-1 immunized mice, levels of murine IgG autoantibodies specific for collagen VII fragments were also measured by ELISA using the recombinant full-length form of the antigen (Fig. 2C). Although the cutaneous manifestations of EBA were restricted to just half of the experimental groups, levels of fragment-specific autoantibodies showed comparable values throughout the 12-week period. In mice immunized with GST-mCVII-3, the autoantibody level was at all times higher, when compared with the other groups (Fig. 2C). The correlation of circulating specific autoantibody levels and the disease activity of individual groups are summarized in Table 3.

**Table 3:** Correlation of serum levels of mCVII fragment-specific mouse antibodies with disease activity

mCVII fragment	Spearman *r*	*P*-value	95% CI	XY pairs
mCVII-1	*r* = 0.5	0.03	0.03981–0.7814	*n* = 19
mCVII-2	*r* = 0.5	0.022	0.06982–0.7814	*n* = 20
mCVII-3	*r* = 0.62	0.0046	0.2166–0.8424	*n* = 19
mCVII-4	*r* = 0.5	0.023	0.06708–0.7803	*n* = 20

### IgG antibodies against various collagen VII fragments show different complement activation capacity *in vivo*

**Fig. 3:**
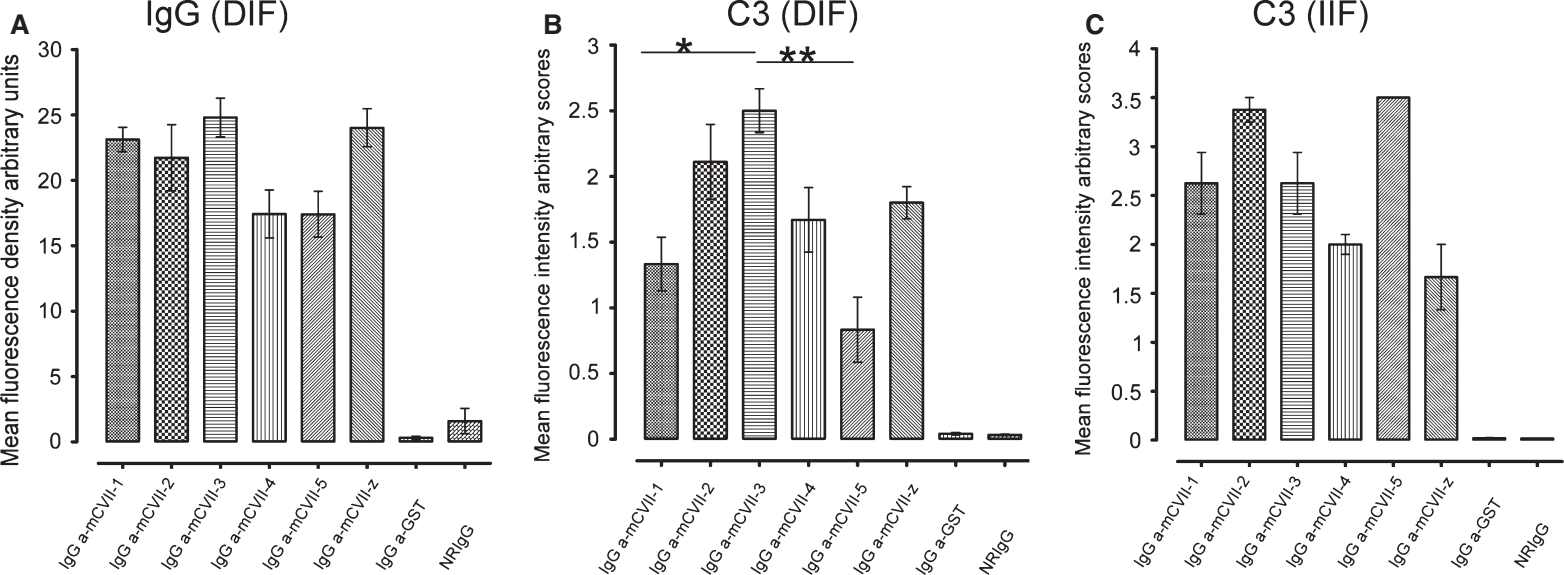
Complement activation triggered by collagen-specific antibodies *ex vivo* and *in vivo*. (**A**) Linear depositions of rabbit IgG at the epidermal basement membrane in frozen sections of perilesional mouse skin biopsies were visualized by direct IF microscopy and the staining intensity measured using ImageJ was expressed as mean fluorescence density arbitrary units ± SEM. (**B**) Linear depositions of murine C3 at the epidermal basement membrane in frozen sections of perilesional mouse skin biopsies were visualized by direct IF microscopy and the staining intensity was semi-quantitatively defined by scoring. Fluorescence intensities were expressed as mean score values ± SEM. Fluorescence intensities were expressed as mean score values ± SEM. *P < 0.05; **P < 0.01, *n* = 6 and were analysed by the Kruskal–Wallis test followed by Dunn's multiple comparison test. (**C**) Complement-binding capacity of murine collagen VII fragment-specific rabbit antibodies was analysed in an *ex vivo* complement binding test. Bound human C3 was visualized by indirect IF microscopy at the dermal–epidermal junction in cryosections of murine skin and the staining intensity was semi-quantitatively defined by scoring.

Immunofluorescence microscopy of perilesional skin from mice receiving collagen VII fragment-specific IgG revealed deposition of mouse complement C3 at the epidermal basement membrane (Fig. S3B of Supporting Information). Although the IgG deposition showed similar fluorescence intensity, significant differences in C3 fixation were seen between antibodies to different fragments (mCVII-3 *versus* mCVII-1 and mCVII-5, *P* < 0.05, *n* = 10, and *P* < 0.01, *n* = 6, respectively), *in vivo* (Fig. 3A and B). Generally, the *in vivo* complement-binding capacity of IgG antibodies specific for different collagen VII fragments paralleled the extent of the induced disease, with the exception of mice treated with rabbit IgG specific for the NC2 domain of murine collagen VII (mCVII-z). There were no deposits of murine C3 at the DEJ of mice injected with anti-GST antibodies or control IgG. To examine the complement activating capacity of rabbit immune sera *ex vivo,* a complement binding test was performed with IF microscopy on frozen sections of normal murine skin. All collagen VII fragment-specific immune sera, but not serum against GST and from normal rabbits, elicited deposition of human C3 at the DEJ, as shown by IF microscopy (Fig. 3C). When scoring the C3 deposits, no significant difference in the *ex vivo* complement-fixing ability of IgG antibodies against different fragments of collagen VII was seen (Fig. 3C).

### IgG antibodies against various collagen VII fragments show different ability to induce neutrophil recruitment *in vivo*, and leucocyte-dependent dermal–epidermal separation *ex vivo*

**Fig. 4:**
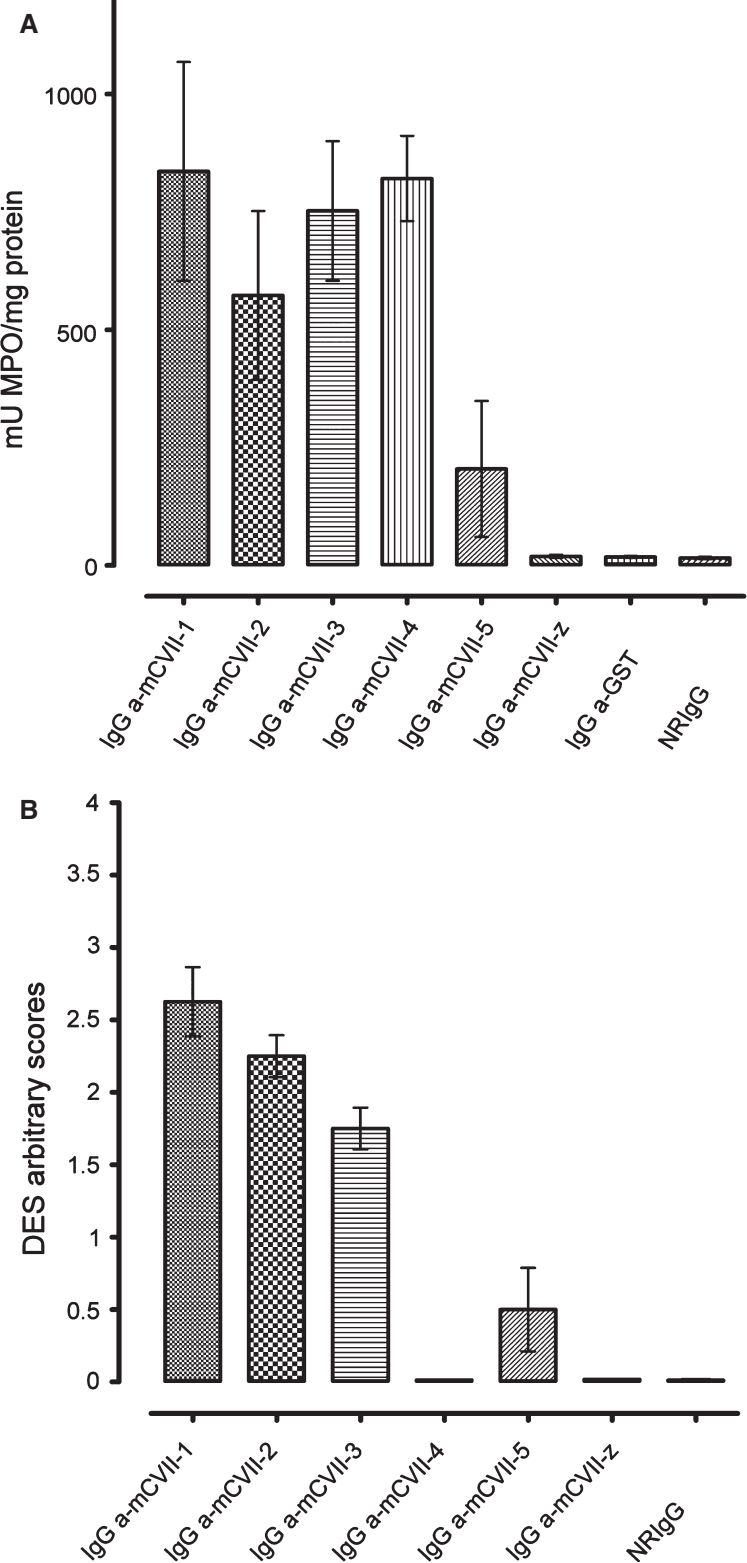
Pathogenic capacity of collagen-specific antibodies. (**A**) Significantly more neutrophil infiltration was evaluated by measuring myeloperoxidase activity in skin biopsies of collagen VII fragment-specific injected mice as compared to NRIgG injected controls; *P* < 0.0001, *n* = 8, Dunnett's multiple comparison test. (**B**) Dermal–epidermal split inducing capacity of rabbit antibodies against fragments of murine collagen VII was assessed, *ex vivo*. Significantly more dermal–epidermal separation occurred in sections incubated with mCVII-1-, mCVII-2- and mCVII-3-specific IgG when compared to the NRIgG incubated ones; *P* < 0.01, *n* = 7, Dunnett's multiple comparison test.

Histological analysis of lesional skin biopsies from diseased mice injected with collagen VII-specific IgG showed subepidermal splits and inflammatory infiltrates, dominated by neutrophils. Neither signs of dermal–epidermal separation, nor leucocyte infiltration were seen in mice receiving antibodies against mCVII-z, GST or NRIgG. To quantify the recruitment of granulocytes *in vivo*, we performed the MPO assay from biopsies of mouse ear. On day 12, the granulocyte infiltration was significantly higher in mice injected with antibodies against mCVII-1, mCVII-2, mCVII-3, and mCVII-4 compared with the groups receiving NRIgG (*P* < 0.0001, *n* = 8; Fig. 4A). A positive correlation between the degree of granulocyte infiltration in the ears of the mice and the disease activity as well as the ear thickness was observed in the different groups (Table 4). Groups injected with mCVII-2- and mCVII-4-specific IgG showed a high degree of correlation in both disease activity and ear thickness, whereas mice receiving antibodies against mCVII-3 had a very high correlation factor as far as the disease activity is considered, but a medium correlation when the ear thickness was examined. To assess the rabbit immune sera's ability to induce granulocyte-dependent dermal–epidermal separation, cryosections of mouse skin were incubated with collagen VII fragment-specific or control sera, and subsequently with human leucocytes. Collagen VII fragment-specific antibodies induced varying degrees of dermal–epidermal separation. Normal rabbit serum as well as antibodies against fragments mCVII-4 and mCVII-z failed to induce dermal–epidermal separation, whereas antibodies against fragments mCVII-1, mCVII-2 and mCVII-3 had the strongest effect *ex vivo* (Fig. 4B).

**Table 4:** Correlation of granulocyte infiltration in the ear and disease activity and ear thickness

	Spearman *r*		*P*-value		XY pairs
		
	Disease activity	Ear thickness	Disease activity	Ear thickness	
IgG α-mCVII-1	*r* = 0.59	*r* = 0.41	0.0013	0.036	*n* = 26
IgG α-mCVII-2	*r* = 0.81	*r* = 0.8	<0.0001	<0.0001	*n* = 26
IgG α-mCVII-3	*r* = 0.97	*r* = 0.5	<0.0001	0.0096	*n* = 26
IgG α-mCVII-4	*r* = 0.97	*r* = 0.86	<0.0001	<0.0001	*n* = 26
IgG α-mCVII-5	*r* = 0.5	*r* = −0.18	0.016	0.399	*n* = 22

### Induced IgG1 and IgG2b autoantibodies against murine collagen VII fragments correlate with disease activity

The IgG subclass levels in sera of SJL-1 mice immunized with different fragments of collagen VII were measured by ELISA. Our results, summarized in Figure S4 of Supporting Information, show a heterogeneous IgG1, IgG2a and IgG2b, but no IgG3 subclass autoantibody response to the full-length form of collagen VII in the mice immunized with individual fragments or equimolar mix of collagen VII fragments. In the control group, no antibodies against collagen VII were detected. The non-complement-fixing IgG1 and the complement-fixing IgG2a and IgG2b levels showed significant differences among mice immunized with collagen VII fragments (Fig. S4 of Supporting Information, lower panel), especially the GST-mCVII-3 group had significantly more circulating specific IgG1, IgG2a and IgG2b antibodies when compared with the GST-mCVII-4 and GST-mCVII-z groups (*P* < 0.0001, *n* = 8). The levels of the measured IgG subclasses for all experimental groups (GST-mCVII-1, GST-mCVII-2, GST-mCVII-3, GST-mCVII-4, GST-mCVII-5, GST-mCVII-z and GST control) correlated with the extent of the skin disease in case of IgG1 (*r*_s_ = 0.875, *P* = 0.0072) and IgG2b (*r*_s_ = 0.875, *P* = 0.0072), but not for IgG2a (*r*_s_ = 0.24, ns).

## Discussion

Antigen-specific T- and B cell-directed immunomodulatory therapies are attractive approaches for autoimmune diseases, because of their putative specificity for the pathogenic process and the promise of a convenient profile of side-effects. The development of these therapies requires the thorough characterization of the pathogenic epitopes. Studies, using EBA patients' sera revealed that the epitopes recognized by their autoantibodies are distributed along the whole collagen VII molecule, still, the majority of antigenic determinants were shown to localize within the NC1 domain [12,28–32]. However, the distribution of pathogenic epitopes on collagen VII has not yet been addressed. In this study, we therefore performed a functional epitope-distribution mapping using the previously established experimental models of EBA. Our results demonstrate that antibodies targeting multiple, distinct epitopes distributed over the entire NC1 domain of collagen VII induce blistering skin disease *in vivo*.

In a first set of experiments, we compared the pathogenic potential of antibodies directed against different fragments of collagen VII, using the passive transfer mouse model of experimental EBA [8]. When injected with antibodies generated against different fragments of collagen VII, mice developed a blistering phenotype. As suggested by descriptive epitope mapping results in EBA patients, we have found pathogenic epitopes to reside within the NC1 domain of collagen VII. Antibodies against different collagen VII NC-1 fragments displayed blister-inducing potential of different degree. Our current results extend our previous data [8] and are in line with the finding that EBA patients' antibodies against the CMP domain of collagen VII [33], which corresponds to the mCVII-1 fragment in our study, are pathogenic *in vivo*. Although IgG antibodies against different fragments of collagen VII were pathogenic *in vivo,* the clinical phenotype they induced was slightly different, depending on the antibody specificity. The observed clinical presentation differences could be because of the heterogeneity in the fine molecular specificity of autoantibodies, which may be an important determinant of the extent and localization of skin lesions and may, at least in part, account for the clinical heterogeneity and presentation of EBA in patients. Further studies should address the correlation of autoantibody specificity with a particular clinical phenotype.

A further relevant aspect is the potential existence of cryptic epitopes on collagen VII. It is likely that polyclonal autoantibodies in EBA patients and in our animal model show different affinity for the epitopes and that some epitopes, which are recognized *in vitro* by immunoassays, are cryptic and masked in the native conformation in the skin. To account for these and other possible differences in their binding to the epidermal basement membrane, we have used antibody preparations adjusted to the same reactivity, as measured by indirect IF microscopy on murine skin sections.

As expected based on the available descriptive epitope mapping data, antibodies against different epitopes within the NC1 domain, but not against the NC2 domain induced skin blistering in the present study. These results strongly suggest that autoantibodies binding to the NC2 domain of collagen VII in EBA patients have a lower pathogenic potential. However, although we have injected IgG preparations showing similar reactivity against the basement membrane, the levels of collagen VII-specific antibodies were lower in the serum of mice treated with IgG specific to the NC2 domain. A likely explanation of this phenomenon is the fact that preparations against mCVII-z contained a lower collagen VII-specific IgG to total IgG ratio which resulted in the injection of higher total amounts of IgG. This could lead to a higher saturation of the neonatal Fc receptor for IgG with subsequent enhanced catabolism of the injected IgG. Therefore, the pathogenicity of NC2-specific autoantibodies needs further investigation using standardized antibodies showing similar binding and effector properties (*e.g*. monoclonal collagen VII-specific antibodies of the same isotype).

A weight loss has been usually observed in mice injected with antibodies against collagen VII [8,38]. A binding of these antibodies to collagen VII expressed in the colon is discussed as the main cause of the weight loss in mice [38,39]. An alternative explanation for this phenomenon is that the weight loss is induced by antibodies against GST present in the IgG preparations. To formally exclude this possibility, in our present study, we injected mice with IgG against GST only. While we have observed a weight loss of different extent in mice injected with rabbit IgG generated against GST-fusion proteins containing sequences of collagen VII NC1, mice injected with rabbit IgG against GST did not show skin disease or weight loss. Although the cause of this phenomenon is not known, it is tempting to speculate that by binding to collagen VII in the intestine, the autoantibodies induce local inflammation resulting in malabsorption [38,39].

An autoimmune response and active subepidermal blistering disease is induced in susceptible SJL mice by immunization with autologous collagen VII [19,40]. We have used this active disease model to complement and extend the functional epitope-distribution mapping by the passive transfer of collagen VII-specific antibodies. Blistering skin disease was induced by immunization with fragments of the NC1 domain of collagen VII, but not with GST alone or with the NC2 domain of collagen VII. These data are generally in agreement with data from the experiments using the passive transfer of collagen VII-specific rabbit IgG. However, similar to previous findings in mice immunized with collagen VII, we did not observe a significant weight loss in mice of these groups [19,41].

Several mechanisms of tissue damage by collagen VII-specific autoantibodies have been proposed [27], which may be classified as relying on the capacity of autoantibodies to activate leucocyte and complement system by their effector Fc portion or pathogenic events triggered by the binding of autoantibodies to their target antigen, independent of their Fc portion. While limited clinical and experimental data suggest that Fcγ-independent mechanisms play a role for skin blistering in EBA, a wealth of experimental evidence convincingly shows that inflammatory mechanisms, including the activation of complement and leucocytes are required for a full-blown generalized disease. Therefore, to investigate if fine molecular specificity of pathogenic antibodies correlates with their Fcγ-dependent effector functions, we have analysed comparatively the complement and leucocyte-activating capacity of antibodies directed to different fragments of collagen VII. Indeed, antibodies against different collagen VII fragments, which bound to a similar extent to the DEJ in the skin of mice, triggered significantly different complement activation *in situ*. While the extent of complement deposits *in vivo* generally correlated well with disease activity in mice treated with collagen VII-specific IgG, activation of complement in an *ex vivo* fixation assay by the same antibodies showed divergent results.

A further pathogenetically relevant effector function of collagen VII-specific autoantibodies is their leucocyte-activating capacity [25,27]. Antibodies to different fragments of collagen VII showed different capacity to recruit granulocytes *in vivo*, as measured by an MPO assay, which correlated well with the extent of the induced skin disease. A noticeable finding was that the NC2 domain-specific antibodies (mCVII-z) in spite of their complement-activating capacity were not able to recruit and activate leucocytes. This phenomenon may be attributed to the fact that the required threshold, enabling downstream inflammatory mechanisms, is not achieved by these autoantibodies [36]. In further experiments, we analysed the activation of leucocytes by collagen VII fragment-specific IgG in an *ex vivo* model of dermal–epidermal separation in frozen skin sections [17,22,25]. Consistently with the *in vivo* data, we have found that IgG against several N-terminal collagen VII fragments induced significantly more subepidermal cleavage compared with IgG against more C-terminal NC1 fragments and NC2 domain. A peculiar finding was the lack of the ability of antibodies against collagen VII fragment 4 to activate leucocytes *ex vivo*, which did not parallel their capacity to induce skin blistering in animals. This finding suggests that leucocyte-dependent mechanisms of tissue damage triggered by antibodies with specificity to this fragment of collagen VII play a secondary role in experimental EBA. Alternatively, activation of the complement, which is not needed for the induction of dermal–epidermal separation in cryosections [37], may play a more important role in the initial phase of the inflammation *in vivo*, as previously described [42]. On the basis of these findings, we can only speculate that, while both complement and granulocytes appear to play a role for the autoantibody-induced skin blistering, the exact mechanisms may be different depending on the fine molecular specificity of the autoantibodies and the pathogenicity *in vivo* may not closely parallel their complement and leucocyte-activating capacity measured *in vitro* [23].

Epidermolysis bullosa acquisita is a clinically heterogeneous disease. EBA patients may present lesions of skin and mucous membranes at different anatomic sites. Previous descriptive epitope mapping studies in EBA patients revealed that the autoantibodies recognize multiple epitopes mainly within the NC1 domain of collagen VII [28,29]. In addition, reactivity to the NC2 and the triple-helical domain in EBA patients has been described [12,30–32]. One possible determinant of the localization and appearance of blistering is the fine molecular specificity of autoantibodies against collagen VII. Our present findings showing that antibodies directed to different epitopes of collagen VII induce partly different clinical phenotype support this hypothesis. Antibodies specific to the NC2 domain of collagen VII, which partly stained the basal keratinocytes did not induce skin disease during the 2-week observation time further emphasizing that the exact binding sites on collagen VII, determine the pathogenic potential of autoantibodies. Pathogenic antibodies in mice are directed against fragments which overlap with the collagen VII fragments recognized by the majority of EBA patients [28].

The development of antigen-specific immunoapheresis and immunomodulatory therapies is the aim of current intensive translational research endeavours. Our present results have important implications for the development of antigen-specific therapeutic approaches in EBA and other diseases associated with autoimmunity against collagen VII. The existence of multiple pathogenic epitopes distributed across at least the main portion of the NC1 domain of collagen VII was clearly documented by the present study, and the ∼200 amino acid long collagen VII fragments used here likely harbour multiple epitopes within their sequence. Therefore, the use of short synthetic peptides for the development of an effective immunoaffinity matrix appears to be strongly limited. Although the production of the full-length or NC1 domain of collagen VII under Good Manufacturing Practice requirements is certainly possible, for the development of such an immunoapheresis matrix, cost-related feasibility issues will come to the fore. Nevertheless, using several overlapping polypeptides expressed in bacteria covering the collagen VII regions containing the pathogenic epitopes may constitute a more cost-efficient alternative.

As with other autoimmune diseases, autoreactive B cells are centrally involved in the EBA pathogenesis in their double role as precursors of the autoantibody-producing cells and as antigen-presenting cells [27,43,44]. Specifically deleting collagen VII-specific B- and plasma cells arguably offers essential advantages over the generally effective, but rather unspecific global B cell depletion using targeting of lineage markers such as CD20 or CD19. Based on our present findings, antigen-containing therapeutic molecules for targeting collagen VII-specific B cells should cover the whole sequence of the NC1 domain to effectively deplete the pathogenic clones. Data from the literature and our present findings suggest that autoreactive T cells in EBA react with multiple epitopes across the NC1 domain of collagen VII [45]. Therefore, for developing effective T cell-targeted strategies to induce tolerance to collagen VII, a full-length or the NC1 domain of the antigen needs to be used.

In conclusion, this article demonstrates that antibodies targeting several, distinct antigenic determinants distributed along the NC1 domain of collagen VII induce skin blistering in experimental animals. The present data have major implications in the development of T- and B cell-specific immunomodulatory therapies in EBA. In addition, these findings will prove a very useful tool in further dissecting the pathomechanisms in antibody-mediated autoimmune disorders.

## Supporting information

**Figure S1** Distribution of the recombinant proteins corresponding to different fragments of collagen VII and their pathogenic potential *in vivo*. **Figure S2** Reactivity and specificity of rabbit antibodies against different fragments of murine collagen VII. **Figure S3** *In vivo* binding and complement activating capacity of mCVII fragment-specific antibodies. **Figure S4** Circulating IgG subclass levels were measured at week 4 and 10 after the first immunization. **Table S1** Summary of clinical and immunopathological findings in mice injected with rabbit antibodies against collagen VII fragments. **Table S2** Summary of clinical and immunopathological findings of mice immunized with collagen VII fragments.
