# Supplementary material for: Blister-inducing antibodies target multiple epitopes on collagen VII in mice
Source: J Cell Mol Med. 2014 Aug 5;18(9):1727–39. doi: 10.1111/jcmm.12338 (PMC4196649; doi:10.1111/jcmm.12338)
Supplement: Supplementary file 1 [file jcmm0018-1727-SD1.docx]

### **Table of content**

Supporting Information Fig 1

Supporting Information Fig 2

Supporting Information Fig 3

Supporting Information Fig 4

Supporting Information Table 1

Supporting Information Table 2

### Supporting Information Fig S1


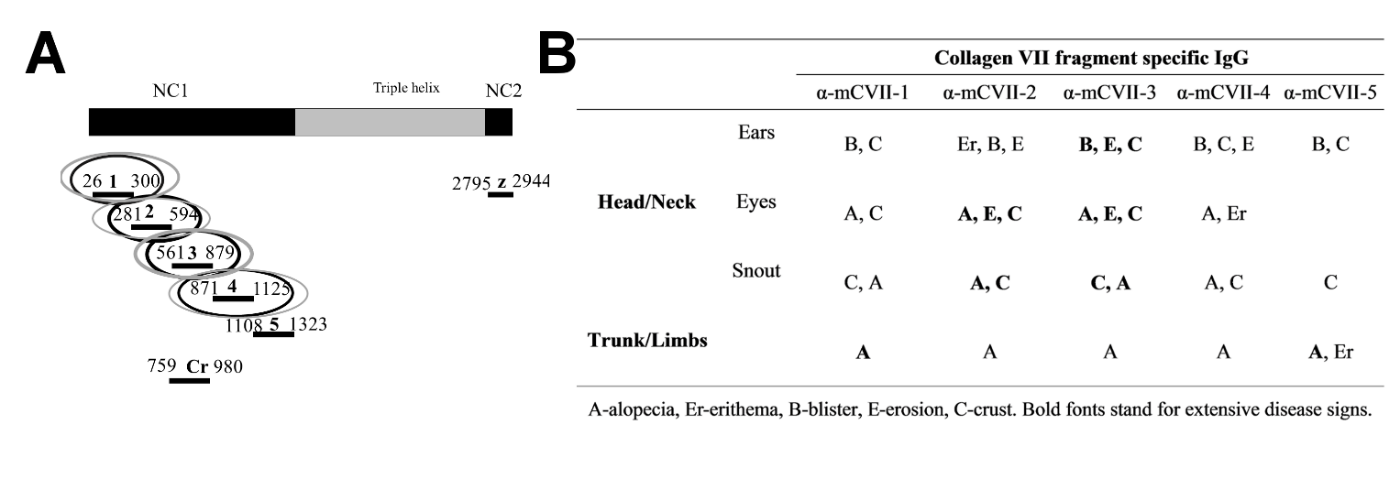


Fig S1: Distribution of the recombinant proteins corresponding to different fragments of collagen VII and their pathogenic potential *in vivo*. **A.** Collagen VII is composed of 3 identical α chains, each consisting of a central triple helical collagenous domain, flanked by a larger amino-terminal noncollagenous domain (NC1) and a smaller carboxy-terminal noncollagenous domain (NC2). The pathogenic potential of antibodies directed against the different fragments of collagen VII are depicted as dark circles and light circles, when using the passive transfer model of experimental EBA and when mice were immunized with the recombinant proteins, respectively. Amino acid residue numbers are shown above the recombinant proteins. **B.** Body-site distribution of lesions in experimental EBA. Bold fonts stand for extensive disease signs.

### Supporting Information Fig S2


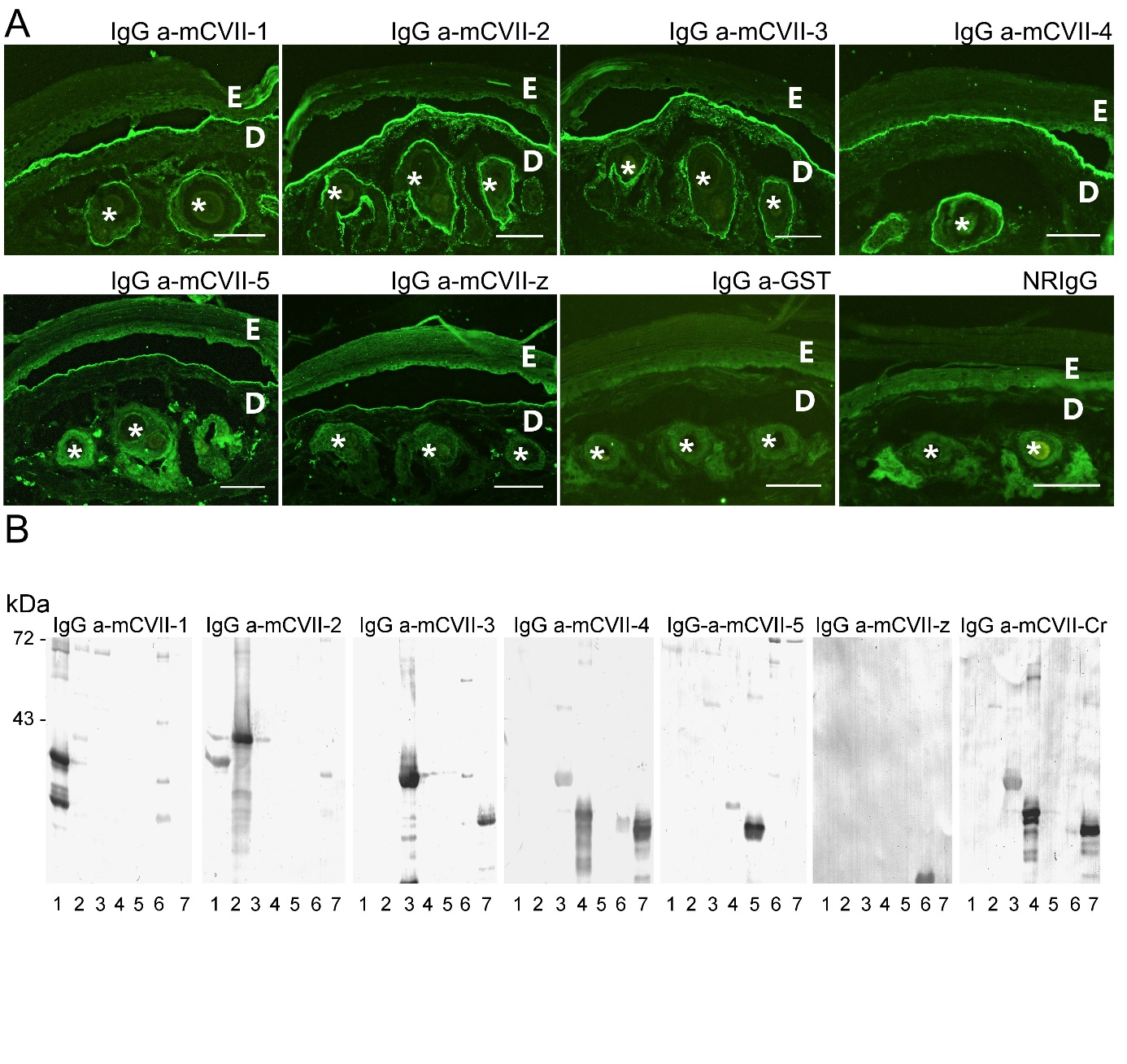


Fig S2: Reactivity and specificity of rabbit antibodies against different fragments of murine collagen VII. **A.** Cryosections of mouse salt split skin were incubated with dilutions of purified IgG from sera of rabbits injected with mCVII-1, mCVII-2, mCVII-3, mCVII-4, mCVII-5, mCVII-z and GST, as well as NRIgG, as control. Linear deposition of rabbit IgG on the dermal side of the split was assessed by IF analysis. E=epidermis, D=dermis, *= hair follicule, scale bar 100 µm. **B.** Equimolar amounts of recombinant proteins His-mCVII-1, His-mCVII-2, His-mCVII-3, His-mCVII-4, His-mCVII-5, His-mCVII-z and His-mCVII-Cr (*lanes 1-7*) were separated by 12% SDS-PAGE and immunoblotted with 200-fold diluted immune rabbit sera. The antibodies recognized specifically the proteins they were raised against: α-mCVII-1 IgG (*lane 1*), α-mCVII-2 IgG (*lane2*), α-mCVII-3 IgG (*lanes 3 and 7*), α-mCVII-4 IgG (*lanes 4 and 7*), α-mCVII-5 IgG (*lane 5*), α-mCVII-z IgG (*lane 6*) and α-mCVII-Cr IgG (*lanes 3, 4 and 7*). mCVII-Cr overlaps with mCVII-3 and mCVII-4.

### Supporting Information Fig S3


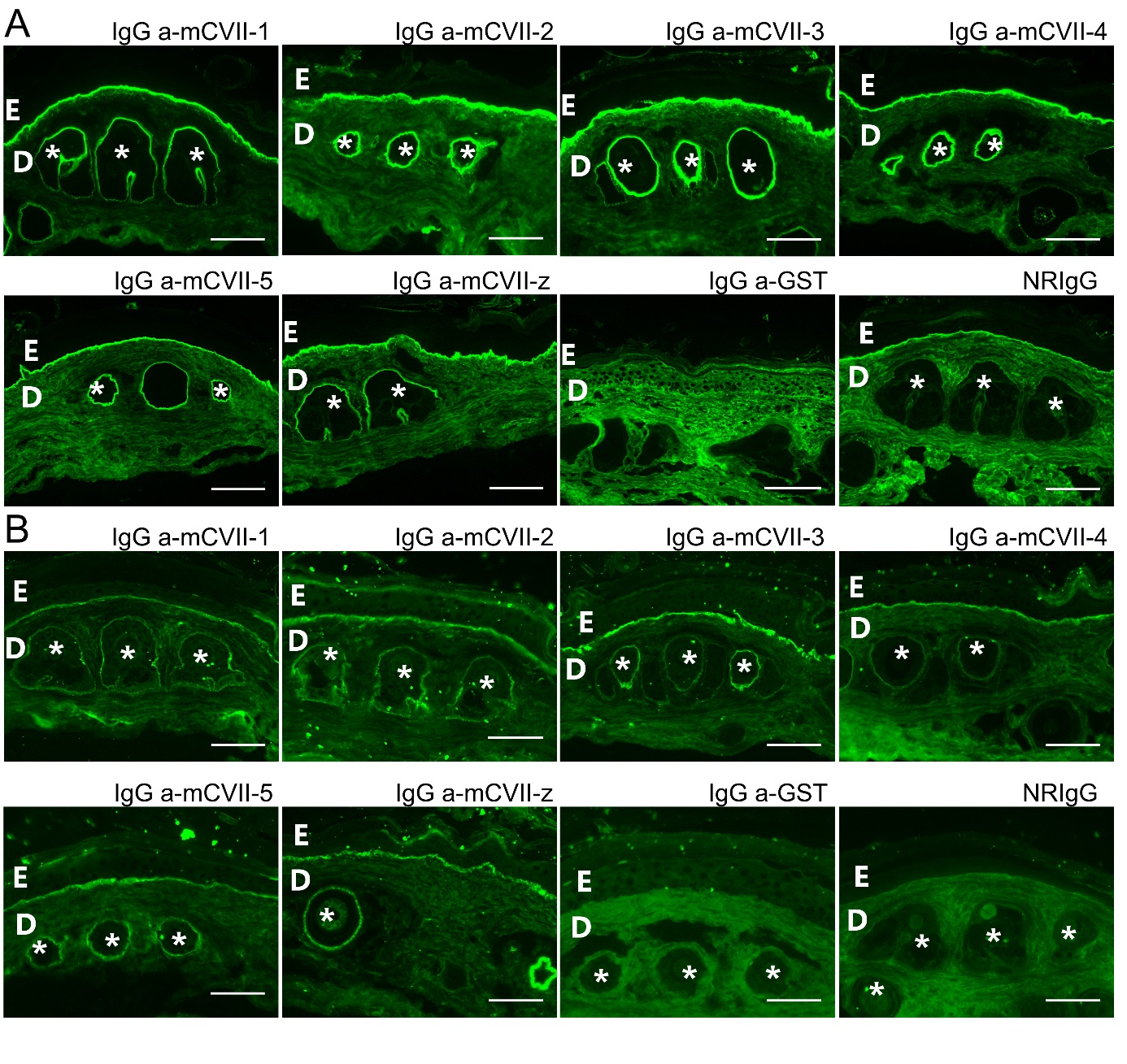


Fig S3: *In-vivo* binding and complement activating capacity of mCVII fragment specific antibodies. **A.** IF microscopy, performed on frozen sections of perilesional mouse skin biopsies reveal linear deposition of collagen VII fragment specific- rabbit IgG. No deposit is seen in the skin of control mice (GST and NRIgG). **B.** Direct IF analysis, performed on frozen sections of perilesional mouse skin biopsies reveal linear deposition of mouse C3. No deposit is seen in the skin of control mice (GST and NRIgG). E=epidermis, D=dermis, *= hair follicule, scale bar 100 µm.

### Supporting Information Fig S4


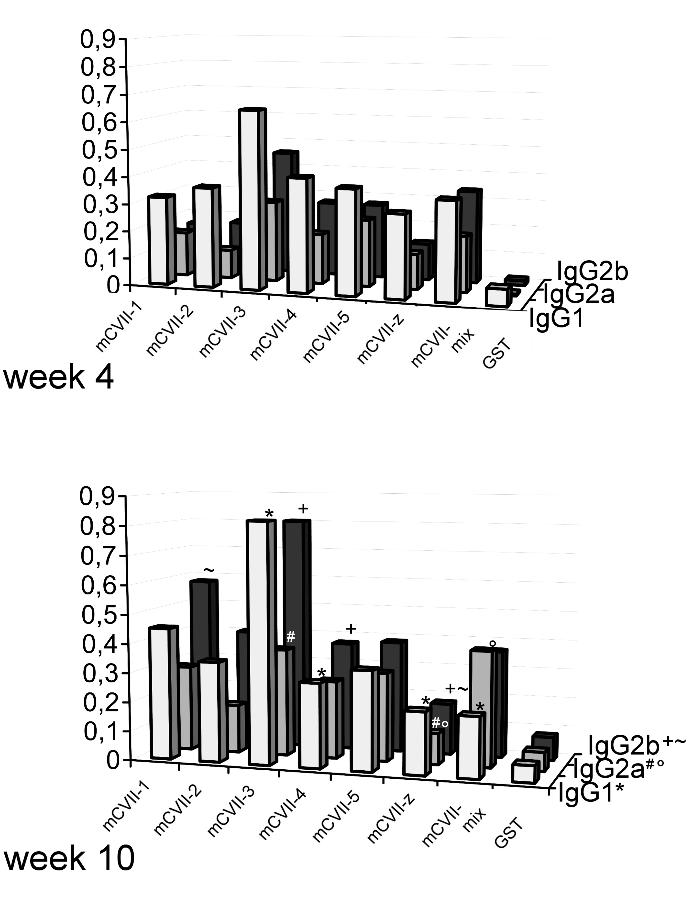


Fig S4: Circulating IgG subclass levels were measured at week 4 and 10 after the first immunization. In sera obtained at week 4 (upper panel) differences in antibody levels of different groups and the control group were seen: significantly more non-complement-fixing IgG1 antibodies were detected in mice immunized with GST-mCVII-3, GST-mCVII-4 and GST-mCVII-5, when compared to the GST controls (p<0.001,n=8); complement-fixing IgG2a antibody levels were significantly higher in GST-mCVII-1, GST-mCVII-3, GST-mCVII-4 and GST-mCVII-5 immunized mice than in control mice (p<0.001,n=8); and the complement-fixing IgG2b antibody levels were significantly different in groups immunized with GST-mCVII-3, GST-mCVII-5 when compared with the control group (p<0.001,n=8). At week 10 (lower panel) IgG subclass level differences among collagen VII fragment immunized groups were seen additionally: IgG1 level was significantly higher in the GST-mCVII-3 immunized mice when compared to the GST-mCVII-4 (p<0.05), GST-mCVII-z (p<0.001) and GST-mCVII-mix (p<0.001) immunized ones; more IgG2a was measured in the GST-mCVII-3 group when compared to the GST-mCVII-z group (p<0.05) and more in the GST-mCVII-mix group when compared with the GST-mCVII-z group (p<0.001); IgG2b levels were significantly different among the GST-mCVII-3, GST-mCVII-4 (p<0.05 ) and GST-mCVII-z (p<0.001) groups and among the GST-mCVII-1 and GST-mCVII-z (p<0.001) groups. (p values calculated based on the Kruskal-Wallis test followed by Dunn's multiple comparison test, n=8 groups; * significant difference in the IgG1 levels, # or ° significant difference in the IgG2a levels, + or ~ significant difference in the IgG2b levels).

### Supporting Information Table S1

Table S1: Summary of clinical and immunopathological findings in mice injected with rabbit antibodies against collagen VII fragments.

|  | **Clinical findings** | **Immunopathological findings** | | **Granulocyte recruitment** | |
| --- | --- | --- | --- | --- | --- |
|  | **Skin lesions** | sera | skin | skin |  |
|  |  | **ELISA^a^** | **DIF (IgG)** | **MPO assay** |  |
| IgG α-mCVII-1 | 10/10 | 9/10 | 7/7 | 6/10 |  |
| IgG α-mCVII-2 | 10/10 | 10/10 | 7/7 | 7/10 |  |
| IgG α-mCVII-3 | 10/10 | 10/10 | 10/10 | 10/10 |  |
| IgG α-mCVII-4 | 10/10 | 10/10 | 10/10 | 10/10 |  |
| IgG α-mCVII-5 | 6/6 | 6/6 | 5/5 | 2/6 |  |
| IgG α-mCVII-z | 0/5 | 5/5 | 5/5 | 0/5 |  |
|  |  |  |  |  |  |

^a^ data taken had an OD>0.1 (490 nm) and were taken from day 4 after the first injection

### Supporting Information Table S2

Table S2: Summary of clinical and immunopathological findings of mice immunized with collagen VII fragments

| **mCVII fragments** | **Clinical findings** | **Immunopathological findings** | | | | | |
| --- | --- | --- | --- | --- | --- | --- | --- |
|  | **Skin leasions** | sera | skin | IgG subclassess^b^ | | | |
|  |  | **ELISA^a^** | **DIF (IgG)** | **IgG1** | **IgG2a** | **IgG2b** | **IgG3** |
| mCVII-1 | 5/10 | 10/10 | 7/7 | 10/10 | 10/10 | 10/10 | 0/10 |
| mCVII-2 | 510 | 10/10 | 6/7 | 10/10 | 7/10 | 10/10 | 0/10 |
| mCVII-3 | 8/10 | 10/10 | 6/6 | 10/10 | 10/10 | 10/10 | 0/10 |
| mCVII-4 | 4/10 | 10/10 | 6/9 | 7/10 | 8/10 | 9/10 | 0/10 |
| mCVII-5 | 0/10 | 10/10 | 0/9 | 6/9 | 7/9 | 8/10 | 0/10 |
| mCVII-z | 0/10 | 10/10 | 0/9 | 8/10 | 4/10 | 7/10 | 0/10 |
| mCVII-mix | 0/10 | 10/10 | 5/5 | 6/10 | 10/10 | 10/10 | 0/10 |

^a^ data taken had an OD>0.1 (490 nm) and were taken from week 8 after the first immunization

^b^ data taken had an OD>0.1 (490 nm) and were taken from week 10 after the first immunization
